# The Role of Antioxidants in Male Fertility: A Comprehensive Review of Mechanisms and Clinical Applications

**DOI:** 10.3390/antiox14081013

**Published:** 2025-08-19

**Authors:** David Bouhadana, Marie-Hélène Godin Pagé, Debbie Montjean, Marie-Claire Bélanger, Moncef Benkhalifa, Pierre Miron, Francis Petrella

**Affiliations:** 1 Division of Urology, McGill University, Montreal, QC H4A 3J1, Canada; 2 Fertilys Fertility Centers, Laval, QC H7S 1Z5, Canadamarie-claire.belanger.chum@ssss.gouv.qc.ca (M.-C.B.);; 3 Centre de Recherche du Centre Hospitalier de l’Université de Montréal (CRCHUM), CHUM Research Center, Montreal, QC J1H 5N4, Canada; 4 Médecine et Biologie de la Reproduction et Laboratoire PERITOX, Université Picardie Jules Verne, CBH-CHU Amiens Picardie, 80054 Amiens, France; 5 Institut National de Recherche Scientifique-Centre Armand-Frappier Santé Biotechnologie, Laval, QC H7V 1B7, Canada; 6 Hôpital Charles-Lemoyne, Department of Urology, Université de Sherbrooke, Greenfield Park, QC J4K 0A8, Canada

**Keywords:** antioxidants, oxidative stress, male infertility

## Abstract

Oxidative stress is a significant factor in male infertility, with increasing evidence evaluating the role of antioxidants in mitigating its detrimental effects on sperm function and quality. This review examines the mechanisms by which reactive oxygen species (ROS) impact male reproductive health. This article synthesizes the different mechanisms at play and highlights key clinical studies published in the literature that contribute to our understanding of antioxidants to treat male infertility. These studies suggest that supplementation with specific antioxidants may improve sperm parameters and increase fertility outcomes, although results vary depending on dosage, duration, and individual health conditions. Despite promising findings, there are inconsistencies across study methodologies and a lack of standardized treatment protocols, which underscore the need for more rigorous clinical trials. Antioxidant supplementation has the potential to serve as a supportive treatment for male infertility, but it should be approached cautiously and appropriately by carefully selecting patients who are deficient in the administered antioxidants. There is a need to better evaluate the long-term impact on reproductive outcomes and determine the optimal supplementation strategies and patient profiles that would benefit most from supplementation regimens.

## 1. Introduction

Infertility is defined as the inability to reproduce after a year of unprotected sexual intercourse. Studies report infertility rates as high as 15%, affecting couples worldwide [1]. Male factor infertility is estimated to account for up to half of these cases. Male factor infertility is increasing in prevalence, as multiple studies have demonstrated declining sperm quality and sperm counts worldwide, making it a significant public health concern [2]. Usually, male factor infertility is due to a modification in either sperm morphology, motility, or concentration found in two sperm analyses that are collected within four weeks of one another [3]. Among men with an abnormal semen analysis, up to 80% of men can have elevated reactive oxygen species (ROS) [4].

ROS are produced by the body’s inability to regulate oxidative stress that results from an imbalance of pro- and antioxidant molecules. ROS are free radicals that usually contain at least one unpaired electron. There are physiological amounts of ROS that allow for several male reproductive functions, such as capacitation and the acrosome reaction [5]. However, the uncontrolled production of ROS can lead to excessive oxidative stress, which can cause a structural modification of the sperm nuclear and mitochondrial genome through the fragmentation of DNA and damage its cell membrane through lipid peroxidation mechanisms. These structural modifications can affect the sperm’s motility and fusion capacity, which can lead to infertility [6]. In addition to having an impact on natural conception, elevated ROS levels can also have detrimental impacts on pregnancy outcomes following assisted reproductive techniques (ARTs) [7].

Antioxidant supplements are thought to improve sperm quality by reducing oxidative stress, which could help prevent the disruption of sperm DNA integrity [8]. While studies have demonstrated improvements in sperm parameters with the use of antioxidants, there are currently no dedicated guidelines elaborating on the use of antioxidants, regulating their use in clinical practice [8,9]. The European and American Urological Association guidelines indicate that, while antioxidants may improve sperm parameters and are not harmful, no conclusive data is available to suggest that they can improve fertility outcomes [10,11]. The benefit of antioxidant supplementation has been studied by many randomized controlled trials; however, these studies do not show consistent results wherein antioxidant supplementation can help improve sperm characteristics but does not always translate to improved pregnancy outcomes. In light of the divergent findings reported, we aim to provide a comprehensive review of the literature related to the mechanisms and clinical applications of antioxidant supplementation in male infertility. The variability in efficacy of antioxidant supplementation warrants further investigations to be able to better understand the patient populations that can benefit most from these therapies. In addition, we hope to help establish a consensus on the criteria for antioxidant supplementation and properly select patients after confirming that they are either deficient in antioxidants or would benefit from their reductive stress potential.

## 2. Oxidative Stress in Male Fertility

**Figure 1:**
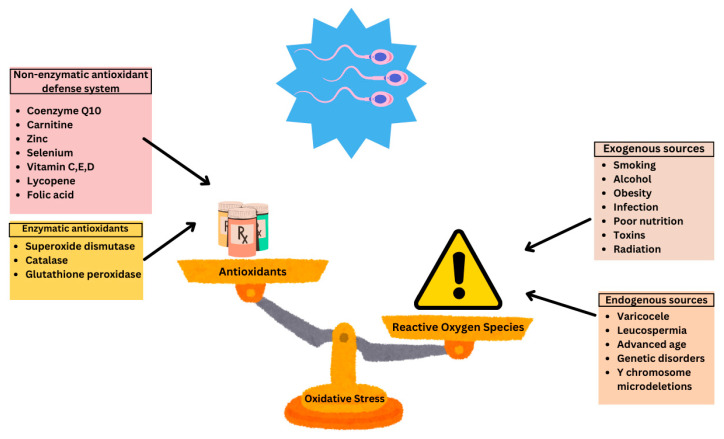
Sources of oxidative stress and antioxidant defense systems in male fertility. Reproduced with permission from Ben Khalifa, *Nutrients*; published by MDPI 2025 [12].

Multiple factors can lead to an increase in oxidative stress including the physiological production of ROS, exogenous sources such as varicocele and infection, as well as environmental and lifestyle factors (Figure 1).

### 2.1. Physiological ROS Production

While there are multiple pathological causes of ROS production, natural biological processes also lead to a physiological amount of ROS being produced, as these allow for adequate spermatozoa maturation when present in ideal concentrations. ROS are mainly produced in spermatozoa via two pathways including the NADPH oxidase in the plasma membrane and the NADH oxidoreductase system in the mitochondria [13]. Mitochondria are particularly abundant within spermatozoa as they require a significant amount of energy to be functionally motile [13]. The production of ROS in regulated quantities is essential for sperm to proceed with important functions including their capacitation and acrosome reaction [5,12]. However, when dysregulated, ROS can be detrimental and affect one’s fertility potential.

### 2.2. Environmental and Lifestyle Factors

There are also predisposing environmental factors that can increase seminal ROS and consequently lead to infertility. Globally, cell phones and electronic gadgets are used every day. Studies have shown that the emission of radiofrequency electromagnetic waves from cell phones may affect sperm concentration, motility, and viability both in vitro and in vivo [14]. Nevertheless, significant heterogeneity remains and the results are inconclusive. These warrant further investigation with better-designed prospective studies [15]. In addition, a recent review article elucidates the impact that environmental pollutants can have on semen quality and male fertility [16]. For instance, there are volatile air pollutants as well as occupational hazards, including pesticides, food additives, and industrial waste products that can affect sperm quality via increased DNA fragmentation and sperm morphological changes [17]. The exact mechanism by which air pollutants can affect fertility outcomes is not fully understood, but is thought to be related to hormonal disruption, the increased production of ROS, and DNA adducts [18]. There is also recent evidence demonstrating that environmental pollutants, mainly heavy metals, such as mercury, can alter protamine-like proteins, which can affect the chromatin structure of sperm and increase oxidative stress [19]. A study carried out by Di Nunzio et al. demonstrated that, among three highly polluted areas in Italy, macro and trace elements were significantly higher in semen than in serum levels, which could suggest that semen can potentially serve as an early marker of environmental toxin exposure [20].

Multiple lifestyle factors can influence the amount of ROS produced. Obese men are three times more at risk of infertility [21]. Studies have shown that an increase in adipose tissue can decrease the levels of serum and free testosterone while promoting levels of estrogen [22]. In addition, obesity also has an impact on the levels of seminal ROS. Excessive adipose tissue can cause a dysregulation of adipocytokines, leading to increased ROS production [23]. A systematic review article revealed that smoking can lead to a decrease in sperm motility and concentration due to the increase in leucocytospermia, which produces an excess of ROS [24]. Other mechanisms that may explain the decrease in sperm function could be related to the decreased expression of the checkpoint kinase 1, which increases sperm cell destruction and decreases sperm quality [25]. It was also shown that smokers may require up to three times the dose of antioxidant supplements to maintain plasma levels comparable to non-smokers [26]. The use of e-cigarettes can also impair male reproductive health as it can disturb the hypothalamo-pituitary axis, increase DNA fragmentation levels, modify the function of the seminiferous epithelium, and ultimately lead to increased apoptosis of spermatogonia and spermatocytes [27,28]. Another lifestyle factor that can affect sperm quality is alcohol. The excessive consumption of alcohol can impair male reproductive hormone production and cause spermatogenic arrest [29]. A study demonstrated that increased alcohol intake led to increased oligozoospermia, which can be suggestive of progressive testicular damage as a result of their increased alcohol intake [30]. Nutrition is another key factor in optimizing fertility. A systematic review conducted by Salas-Huetos et al. demonstrated that a diet including many sources of antioxidants and omega-3 fatty acids, such as the Mediterranean diet, can help improve semen quality and have a protective effect against environmental pollutants. Conversely, they also indicate that diets rich in processed meat, alcohol, sugar-sweetened beverages, and soy foods could negatively affect semen quality [31].

Excessive heat to the scrotum is another risk factor for male infertility. This heat stress can lead to an increase in oxidative stress, sperm DNA damage, germ cell apoptosis, and even spermatogenic arrest [32]. Many sources of genital heat stress can lead to impaired sperm parameters. For one, varicoceles can lead to the increased production of ROS. A varicocele is the development of an abnormal dilation of the testicular pampiniform plexus due to the stasis and backflow of venous blood, likely due to a dysfunction in venous valves. Varicoceles may often go undiagnosed and not cause any issues, as up to 65% of men with varicoceles may have normal semen parameters [33]. However, reports note that its prevalence among infertile patients can be as high as 40% [34]. A varicocele can lead to excessive temperatures in the scrotum, which can consequently hinder the development of functional sperm, testicular histology, and productivity of reproductive hormones and increase the production of ROS [33,35,36]. While the exact cause of infertility secondary to varicoceles is not completely understood, the leading theory explaining the selective infertility of varicoceles is thought to be related to increased levels of oxidative stress [37]. Studies explain that the increased hydrostatic pressure produced on the walls of the veins can lead to an increased production of ROS, wherein higher varicocele grades have higher levels of oxidative stress [38,39]. An increase in oxidative stress results from the increase in scrotal temperature, hypoxia, and reflux of toxic metabolites from the adrenals and kidneys [40]. Other mechanisms by which varicocele may lead to infertility include its negative effect on testosterone production and the increased production of ROS from mitochondrial membranes due to heat stress [40,41].

### 2.3. Mechanisms of Oxidative Damage

**Figure 2:**
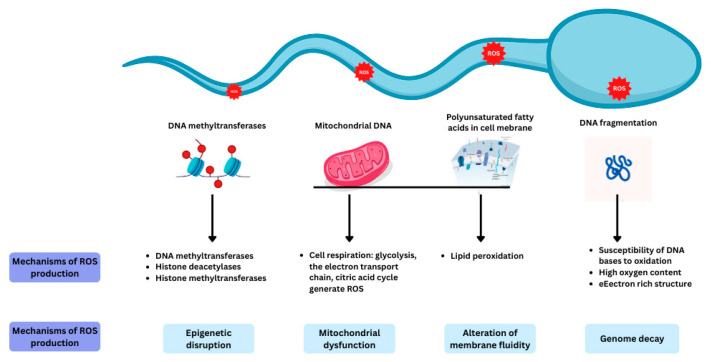
The mechanisms involved in ROS production within spermatozoa. Reproduced with permission from Ben Khalifa, *Nutrients*; published by MDPI 2025 [12].

Spermatozoa are more vulnerable to oxidative stress compared to other cells in the body as they contain large quantities of polyunsaturated fatty acids in their plasma membranes and have a limited ability to eliminate ROS. The different mechanisms at play in producing ROS within the spermatozoa are highlighted in Figure 2.

#### 2.3.1. Sperm Membrane Lipid Peroxidation

The sperm plasma membrane is composed of multiple polyunsaturated fatty acids that facilitate the fusion of membranes. This is an important characteristic of sperm that promotes the acrosome reaction. However, this also renders sperm susceptible to a cascade of free radical production that can lead to increased oxidative stress [42]. The sperm membrane constituents facilitate sperm membrane lipid peroxidation, which further produces free radicals like hydrogen peroxide. Increased levels of hydrogen peroxide can lead to the depletion of intracellular adenosine triphosphate (ATP) and potentially lead to cell death [43]. The increased production of free radicals can directly damage sperm DNA and the integrity of the sperm’s plasma membrane. As a result, the sperm’s ability to fuse with an oocyte is significantly affected [44].

#### 2.3.2. DNA Fragmentation

DNA fragmentation results from increased oxidative stress and consequently leads to an increase in ROS that can oversaturate the sperm cell’s few antioxidant defense mechanisms and increase oxidative stress, which further directly damages DNA [45]. DNA fragmentation occurs via the oxidation of DNA base pairs, single or double-stranded DNA breaks, chromatin decondensation or through the activation of caspases and endonucleases. As a result of this DNA damage, the mitochondria can be affected and generate additional ROS due to the dysfunction of their electron transport chain leading to increased electron leakage. While it is true that sperm with elevated levels of DNA fragmentation do not lose their ability to fertilize oocytes, the damaged nuclear DNA can lead to errors that are carried over to the development of the embryo [46]. The DNA fragmentation index (DFI) is a helpful tool to clinically determine which patients are at risk of infertility. Studies previously used a cutoff of 30–50% to indicate a higher chance of infertility and pregnancy loss [47,48]. Four different assays can reliably measure the DFI. These include the Sperm Chromatin Structural Assay (SCSA), Terminal deoxynucleotidyl transferase dUTP nick end labeling (TUNEL), Comet, and Sperm Chromatin Dispersion (SCD) assays [49]. The SCSA is the gold standard for DFI measurement and relies on measuring levels of acid denaturation at areas of DNA strand breaks using flow cytometry. The other listed methods use different markers for DNA damage including the 3′-hydroxyl terminal deoxynucleotidyl transferase enzyme found on the free ends of DNA breaks, the production of chromatin halo following DNA denaturation, and the measurement of protein depletion using single-cell electrophoresis [50]. In addition to its effect on sperm parameters, DNA fragmentation has also been shown to negatively affect clinical fecundity and outcomes following assisted reproductive therapy, increasing the risk of pregnancy loss [51].

#### 2.3.3. Mitochondrial Dysfunction

The increase in ROS can also damage mitochondrial DNA. Sperm are abundant in mitochondria as they require significant energy sources to ensure their motility. The sperm mitochondria and lipid membrane are the two main regions where excessive ROS are produced [52]. Sperm mitochondrial DNA is more susceptible to damage as its circular nature consists of fewer DNA base pairs and has no repair mechanisms to counterbalance ROS damage. In addition, the mitochondrial membrane is also a significant source of ROS; therefore, if it is damaged, it can in turn increase the production of ROS and lead to more oxidative stress [4,53].

#### 2.3.4. Sperm Methylation

Oxidative stress can also result from epigenetic changes such as DNA methylation as the transcription of genes responsible for the sperm’s endogenous antioxidant defense system can be hindered, making them more vulnerable to ROS. It is hypothesized that epigenetic changes mainly occur in the epididymis during sperm maturation. An increase in ROS can lead to hypomethylation of sperm DNA, which can reduce spermatogenesis and precipitate the development of testicular cancer [54]. Changes in DNA methylation patterns can even lead to altered testicular histology [55]. An impaired folate and homocysteine metabolic pathway is a suspected mechanism that can lead to hypomethylation, as it is responsible for producing methyl donors that are crucial in facilitating DNA methylation [56]. DNA methylation is an important factor for ensuring both healthy sperm function and embryo development [46,57]. Indeed, studies have shown that hypomethylation of sperm DNA often correlates with oligozoospermia, failure with in vitro fertilization (IVF), and can also lead to poor embryo quality. This demonstrates that DNA methylation can serve as a potential biomarker for sperm that are susceptible to oxidative stress, which can be identified through the use of methylation screening panels [58].

### 2.4. Oxidative Stress Markers in Male Infertility

Oxidative stress can result following any of the mechanisms described above. While it can be more difficult to measure these values accurately, they can all be measured using a combination of different techniques [59]. To be able to measure the production of ROS, studies often use flow cytometry in combination with fluorescence or chemiluminescent assays to detect levels of commonly produced ROS, such as hydrogen peroxide, a hydroxyl radical, and a peroxynitrite anion [60]. Levels of sperm membrane peroxidation can also be measured by a fluorescent response using the kit BODIPY C11 test (Invitrogen, Waltham, MA, USA), which identifies cells wherein oxidative degradation of lipids occurs [61]. The CellROX^TM^ system is another method that is currently under investigation in the clinical setting [62,63]. This system uses different fluorescent probes to collect precise and direct measurements of ROS found in sperm. For instance, the MitoSOX Red probe (Invitrogen, Waltham, MA, USA) can be used to measure oxidative stress localized in the mitochondria, whereas the CellROX Green (Invitrogen, Waltham, MA, USA) and Deep Red probes (Invitrogen, Waltham, MA, USA) can be used to detect intracellular ROS. This system offers an advantage compared to the traditional kit BODIPY C11 assay, which can only be performed using flow cytometry, as it can use spectrophotometry, fluorescence microscopy, and/or flow cytometry [64]. This makes the measurement of ROS more accessible for centers that do not have access to a flow cytometer, which is not widely available and has a large maintenance cost. While this system is promising for measuring oxidative stress in spermatozoa, further studies are needed to evaluate its potential in identifying patients who may benefit most from antioxidant supplementation.

There are two other commonly used assays to measure levels of oxidative stress, and each has its benefits and disadvantages. These include the Luminol and MiOXSYS assays [65]. The Luminol system relies on chemiluminescence as it measures the intensity of light that is produced when a luminescent probe reacts with the free radicals in the system. This tool was used to help distinguish between fertile and subfertile men undergoing a vasectomy with a specificity and sensitivity of 76% and 73%, respectively [66]. Multiple studies have attempted to develop cut-off values using relative light units as a measure; however, these varied significantly, especially in patients with leucocytospermia [66,67]. This system was also used to measure oxidative stress among patients undergoing ARTs and noted that increased ROS levels correlated with poor pregnancy outcomes for couples undergoing IVF but did not help predict pregnancy outcomes for patients undergoing intracytoplasmic sperm injections (ICSIs) [68]. However, these studies are limited by their small sample size and failure to adjust for baseline characteristics.

More recently, the MiOXSYS system was developed to measure levels of seminal oxidative stress [69]. This system measures oxidative stress by detecting the electron transfer that occurs in real time to calculate the oxidation–reduction potential. This is a cost-effective and reproducible technique that can be safely implemented in the clinical setting but is limited in its ability to differentiate between different ROS radicals [70,71,72]. In a large multicentric trial, the MiOXSYS system was successful in identifying infertile men with abnormal semen parameters with a sensitivity and predictive value of 98% and 94%, respectively [70]. Studies have also shown that the MiOXSYS system can be useful in predicting pregnancy outcomes for couples undergoing both IVF and ICSI [73,74]. Multiple studies have evaluated this system; however, these studies demonstrate that the MiOXSYS system is not as useful in the clinical setting for several reasons. For one, sperm static oxidoreduction potential (sORP) index values vary among men with different sperm parameter anomalies and are highly dependent on sperm concentration [72]. This can lead to significant bias and makes it less applicable for oligozoospermic men as this would lead to inaccurate sORP values. In addition, there are no consistent associations between elevated sORP and key markers of sperm quality such as DNA fragmentation or chromatin integrity, which may suggest that sORP may not be a useful marker. In a study conducted at our center, we found that the values obtained using the MiOXSYS system primarily reflect the redox status of the seminal fluid rather than the sperm cells themselves [72]. This has several implications when undergoing ARTs, as isolated spermatozoa are used for insemination; therefore, information generated from the measurement of seminal plasma is not as useful. Given the latter, this makes it very difficult to determine a consensus on a cutoff range that can be applied in the clinical setting. As a result, we would not support the clinical use of MiOXSYS as a standalone diagnostic tool for measuring oxidative stress in sperm. While levels of oxidative stress may be more informative than sperm parameters, there are currently no validated or recommended assays to measure ROS, and the results need to be interpreted with caution [65,71]. The measurement of ROS is restricted to patients who either have an abnormal spermogram, reduced fertilization and embryo development when undergoing ARTs or repeated implantation failure as the system can generate false positive results [75]. This highlights the need for improved methods to measure and quantify oxidative stress including male oxidative stress infertility biomarkers such as 8-hydroxy-2′-deoxyguanosine, the use of omics studies, and DNA methylation panels [76,77].

## 3. Endogenous and Exogenous Antioxidant Defense Systems

The male reproductive system relies on a multi-layered antioxidant defense network to counteract ROS and maintain redox balance. This system comprises endogenous mechanisms produced within the body and exogenous antioxidants acquired from diet or supplements. Together, they work synergistically to protect spermatozoa from oxidative damage, preserve DNA integrity, and support mitochondrial and membrane function. The male reproductive system maintains sperm function and integrity through a finely tuned balance between ROS and antioxidants. Antioxidants in seminal plasma and spermatozoa are broadly categorized into enzymatic and non-enzymatic systems. Table 1 summarizes the different antioxidants described and their mechanism of action in protecting against oxidative stress.

### 3.1. Endogenous Antioxidant Mechanisms

To ensure optimal sperm cell function, maintaining a balanced redox potential, specifically by regulating ROS with antioxidants, is essential. Seminal fluid is naturally rich in antioxidants and also provides nutritional and protective support to sperm [78]. Endogenous antioxidants fall into two categories: enzymatic and non-enzymatic systems. The enzymatic system includes glutathione peroxidase, superoxide dismutase, and catalase, which are all enzymes naturally present in sperm or seminal plasma. In contrast, the non-enzymatic system comprises various compounds obtained through diet, including dietary supplements [78].

#### 3.1.1. Enzymatic Antioxidants

Superoxide dismutase (SOD) is one of the most essential antioxidant enzymes, catalyzing the dismutation of superoxide anions into hydrogen peroxide and oxygen. Isoforms of SOD are located in different parts of the sperm cell. Copper/zinc-containing SOD (Cu/Zn-SOD) is primarily found in the cytoplasm and accounts for the majority of SOD activity, while manganese-containing SOD (Mn-SOD) is located in the mitochondrial matrix. An extracellular form of SOD, also containing copper and zinc, is found in seminal plasma [78]. Studies have indicated that both intracellular and extracellular SOD isoenzymes are predominantly derived from the prostate [79,80].

Catalase (CAT) works in tandem with SOD by converting the hydrogen peroxide produced during SOD activity into water and oxygen. This enzymatic process is crucial, as the accumulation of hydrogen peroxide can be damaging to sperm cells. CT contains a heme group with a central iron atom that facilitates its catalytic function. It has been identified in several cellular locations, including peroxisomes, mitochondria, the endoplasmic reticulum, and the cytosol [81,82]. In the male reproductive system, CT activity has been detected in human sperm as well as in seminal plasma, where its presence is primarily attributed to prostatic secretions [79].

Glutathione peroxidase (GPx) also catalyzes the reduction of hydrogen peroxide and other substrates such as organic peroxides in semen. Its activity depends on the presence of selenium, specifically in the form of selenocysteine located at the enzyme’s active site [80]. Within sperm cells, GPx is primarily located in the mitochondrial matrix, where it protects mitochondrial function from oxidative damage [83]. Interestingly, a distinct nuclear form of GPx has also been identified. This variant plays a protective role in maintaining sperm DNA integrity and is thought to contribute to chromatin condensation during sperm maturation [84]. Furthermore, the presence of GPx in seminal plasma suggests that the prostate may also serve as a source of this enzyme [85].

SOD is the first line of defense against ROS in sperm cells, catalyzing the dismutation of superoxide radicals into hydrogen peroxide and oxygen. Cu/Zn-SOD is primarily cytosolic, Mn-SOD is mitochondrial, and extracellular SOD is present in seminal plasma. These isoforms mitigate the mitochondrial ROS burden, a major source of oxidative stress in spermatozoa [66,67]. Working in synergy with SOD, catalase detoxifies the hydrogen peroxide produced, converting it into water and oxygen. Its localization in peroxisomes and cytoplasm of sperm cells allows detoxification of peroxides throughout the cell, thus limiting membrane lipid peroxidation and DNA damage [68]. To further fortify this enzymatic defense system, GPx comes into play. GPx uses GSH to reduce hydrogen peroxide and lipid peroxides. The mitochondrial isoform protects energy production machinery, while the nuclear isoform participates in chromatin condensation and DNA protection during spermatogenesis [69,83].

#### 3.1.2. Non-Enzymatic Antioxidants

Non-enzymatic antioxidants play a crucial role in supporting enzymatic defenses by directly neutralizing free radicals and helping to maintain redox homeostasis within sperm cells. Seminal plasma is particularly rich in these antioxidants, specifically glutathione, coenzyme Q^10^, zinc, and selenium, providing essential protection against oxidative damage and preserving sperm function.

Glutathione (GSH) is a tripeptide composed of cysteine, glycine, and glutamine. It is a central non-enzymatic antioxidant in sperm cells that exists in reduced (GSH) and oxidized (GSSG) forms, cycling between the two via the glutathione peroxidase/reductase system, with support from NADPH. This cycle enables the neutralization of hydrogen peroxide and lipid peroxides [86]. Clinical studies have shown that glutathione supplementation can improve sperm quality in men with conditions such as varicocele or genital tract inflammation [87].

Coenzyme Q^10^ (CoQ^10^) plays a dual role in the male reproductive system, functioning as both a component of the mitochondrial electron transport chain and a lipid-soluble antioxidant. It is particularly abundant in the mitochondria of sperm, where it supports ATP production necessary for motility. Due to its role in enhancing energy metabolism, CoQ^10^ is often considered beneficial for improving sperm motility and protecting against oxidative damage [88]. Notably, CoQ^10^ helps prevent superoxide generation, thereby reducing oxidative stress-related impairments in sperm function. Studies have shown a significant inverse relationship between CoQ^10^ concentrations and hydrogen peroxide levels, along with a direct correlation between CoQ^10^ and both sperm count and motility [89].

Zinc, an essential trace mineral acquired through diet and supplements, is vital for numerous cellular functions. In addition to supporting DNA stability and maintaining membrane integrity, zinc plays a key role in male reproductive health indirectly through its role as a structural component of antioxidant enzymes [90,91]. Research indicates that zinc helps preserve sperm structure and function, particularly by reducing oxidative stress, cell death, and DNA fragmentation in men with asthenozoospermia [92]. Furthermore, studies have highlighted the protective potential of zinc oxide nanoparticles in cryopreservation media, showing reduced cellular damage during freeze–thaw cycles [93].

Similarly, selenium is a crucial trace mineral that enhances antioxidant defense by serving as an essential component of various selenoenzymes. Its role is closely tied to glutathione activity, supporting cellular redox balance. Over 25 different selenoproteins have been identified, including phospholipid hydroperoxide glutathione peroxidase (PHGPx) and sperm capsular selenoprotein glutathione peroxidase, both of which contribute to the structural stability and function of sperm cells [92]. Insufficient selenium levels have been linked to abnormalities in the sperm midpiece and reduced motility, indicating its fundamental role in maintaining reproductive health [94].

Complementing enzymatic defenses, GSH plays a pivotal non-enzymatic role in ROS neutralization. The GSH/GSSG cycle, mediated by glutathione reductase and fueled by NADPH, helps maintain the redox environment of sperm cells. This system protects thiol groups in proteins and stabilizes mitochondrial and nuclear integrity [70]. In parallel, CoQ^10^ contributes to both energy metabolism and antioxidant defense. Located in the inner mitochondrial membrane, it transfers electrons during oxidative phosphorylation and prevents mitochondrial ROS generation. It stabilizes membrane potential and supports ATP synthesis, indirectly enhancing sperm motility and viability [71]. Trace elements also play vital roles in redox regulation. Zinc stabilizes sperm chromatin and prevents apoptosis by modulating caspase activity. Additionally, selenium, through its incorporation into selenoenzymes such as GPx and sperm capsular selenoproteins, protects membranes from oxidative damage and contributes to the structural architecture of mature sperm [72,78].

### 3.2. Exogenous Antioxidants

Dietary antioxidants from fruits, vegetables, fish, and nuts bolster endogenous defense systems by supplying vitamins, minerals, and phytochemicals that counter ROS generation and lipid peroxidation.

Among these, vitamin C (ascorbic acid) is a water-soluble antioxidant whose concentration in seminal plasma is significantly higher compared to that in blood serum, suggesting its essential protective role in the male reproductive tract. It acts by neutralizing ROS such as hydroxyl radicals, superoxide anions, and hydrogen peroxide, thus minimizing endogenous oxidative damage. Supplementation with vitamin C has been associated with reduced sperm DNA fragmentation in both normozoospermic and asthenozoospermic men, and clinical administration at a dose of 1000 mg/day has demonstrated improvements in sperm concentration, motility, and morphology.

Vitamin E (α-tocopherol) is a fat-soluble antioxidant concentrated in cellular membranes that complements vitamin C’s action by interrupting lipid peroxidation chains initiated by ROS. Higher seminal levels of vitamin E are positively correlated with improved sperm motility. The supplementation of vitamin E has been shown to reduce lipid peroxidation and potentially improve pregnancy rates in cases of asthenozoospermia. Moreover, co-administration with selenium has synergistic effects, further enhancing sperm quality. Together, vitamins C and E protect DNA, acrosomal enzymes, and the structural components essential for motility [74,76].

Vitamin D is another water-soluble vitamin traditionally known for its role in calcium metabolism. It has recently been implicated in male fertility as well. While its precise antioxidant capacity remains under investigation, it contributes to the regulation of sperm motility and function, possibly through the modulation of intracellular calcium homeostasis. Vitamin D receptors and metabolizing enzymes have been identified in male reproductive tissues, suggesting its local action in spermatogenesis and sperm maturation. Some studies have associated higher serum vitamin D levels with improved semen parameters and testosterone concentrations, indicating a broader regulatory role in reproductive endocrinology [77].

Minerals also play an indispensable role in antioxidant defense. Zinc and selenium act as co-factors for antioxidant enzymes, supporting their catalytic functions, while copper, although essential for SOD activity, becomes pro-oxidant when unregulated. Adequate dietary intake of these trace elements ensures optimal enzymatic activity and mitigates ROS-induced damage such as DNA fragmentation [79,80].

Beyond vitamins and minerals, other dietary compounds enhance oxidative protection through distinct mechanisms. For instance, lycopene is a potent singlet oxygen quencher that integrates into sperm membranes, reducing lipid peroxidation caused by environmental and lifestyle stressors [68]. Its ROS-scavenging capacity helps preserve sperm function and structural integrity. Similarly, carnitines contribute by shuttling long-chain fatty acids into mitochondria, supporting oxidative metabolism and enhancing sperm motility. Their concentration in the epididymal tail is markedly higher than in blood plasma, reflecting their active transport and importance in sperm viability and motility. Their antioxidant effects result from preserving mitochondrial function and limiting ROS leakage [70,82]. Finally, omega-3 fatty acids, especially eicosapentaenoic acid (EPA) and docosahexaenoic acid (DHA), are polyunsaturated fats essential for maintaining the structural integrity and fluidity of sperm membranes. Their downstream metabolites also exert anti-inflammatory and antioxidative effects, further enhancing reproductive outcomes. Increased dietary intake or supplementation with omega-3s has been linked to improved sperm count, motility, and morphology, likely due to their role in stabilizing membranes and supporting mitochondrial function [72].

## 4. Clinical Evidence for Antioxidant Supplementation

The overproduction of ROS generates increased oxidative stress, which can lead to male infertility through different pathways. To help counterbalance this increase in oxidative stress, many studies have examined the use of exogenous antioxidant supplementation to help improve sperm parameters and male fertility. Studies have examined the benefits of different non-enzymatic antioxidants, including CoQ^10^, carnitine, zinc, and selenium, vitamins C, E, and D, lycopene, and folic acid, to evaluate their effect on sperm concentration, motility, morphology, and levels of DNA fragmentation (Table 2) [8].

A systematic review conducted by Ross et al. found that 82% of the included studies improved either sperm parameters or pregnancy outcomes and 60% of the studies improved overall pregnancy rates [104,105]. This improvement was also noted in another review focusing on outcomes following ARTs. More recently, a Cochrane meta-analysis demonstrated that antioxidant supplementation can increase the chance of a live birth [8]. They report an increase of 3 and 2-fold for the odds of pregnancy and live birth, respectively. However, the results of this analysis also included substances with antioxidant properties, studies wherein couples were undergoing IVF, and studies that did not have systematic follow-up [106,107]. There are also studies that did not report these benefits. For example, the Males, Antioxidants, and Infertility (MOXI) multicentric randomized clinical trial demonstrated that the use of antioxidant supplementation did not improve male fertility [108]. In this study, the authors evaluated the use of an antioxidant formulation including vitamin C, E, selenium, carnitine, zinc, lycopene, folic acid, and vitamin D in comparison to a placebo formulation. They showed that, among 144 couples, there were no significant changes in sperm concentration, morphology, motility, and DNA fragmentation between the two groups at three months of treatment. Similarly, other high-quality studies conducted by Balercia et al. and Blomberg Jensen et al. did not show an improvement in fertility outcomes with the use of antioxidants [109,110].

### 4.1. Coenzyme Q^10^

CoQ^10^ is an antioxidant that plays an important role in mitochondrial energy metabolism. Multiple studies have evaluated the role of this antioxidant in improving sperm parameters [111,112,113]. However, these studies were heterogeneous and had small sample sizes. In 2013, Lafuente et al. conducted a systematic review of double-blind randomized clinical trials. They found that CoQ^10^ led to a significant difference in sperm concentration and total sperm motility but did not increase pregnancy rates [95]. These results further demonstrate the role of CoQ^10^ in mitochondrial metabolism, which is an important driver for sperm motility. Of note, one of the trials was specific to men with idiopathic asthenozoospermia, whereas the other two studied infertile men with idiopathic oligoasthenoteratozoospermia. These studies reported pregnancy rates as an outcome, but none evaluated live birth rates as an outcome. In 2021, Salvio et al. conducted another systematic review to evaluate the use of CoQ^10^ alone and in combination with other supplements [88]. They found that CoQ^10^ monotherapy led to an improvement in sperm motility in all but one of the included studies [113]. They also demonstrated an overall improvement in sperm concentration. These results were also noted when CoQ^10^ was used in combination with other compounds. However, it is important to mention that the studies included doses of CoQ^10^ that varied from 20 to 200 mg per day. Therefore, it is unclear what the ideal recommended dosage of CoQ^10^ should be when used in combination with other supplements. Within this systematic review, four different studies demonstrated an improvement in DFI with CoQ^10^ supplementation [114,115,116,117]. For one, Arafa et al. noted an improvement of 9% in DFI with the use of an antioxidant formulation containing 200 mg of CoQ^10^ [115].

### 4.2. Carnitine

Carnitine is an antioxidant that primarily accumulates in the epididymis and can help promote sperm motility and maturation [118]. Carnitine is responsible for transporting key molecules that play an important role in metabolizing fatty acids in mitochondria. A recent randomized controlled clinical trial conducted by Lahimer et al. demonstrated a significant impact on sperm parameters after 3 months of supplementation with carnitine [96]. They used a molecule that was primarily composed of 220 mg of carnitine and was administered four times a day. While no significant difference was noted in conventional sperm parameters including sperm motility, concentration, and vitality, they found that levels of DNA fragmentation decreased from 20% to 14.5%. In addition, this study evaluated pregnancy outcomes and found that antioxidant supplementation improved the pregnancy and live birth rates by 11% and 8%, respectively. A meta-analysis of 23 randomized controlled trials found that carnitine significantly improved sperm motility (7%), sperm concentration, and morphology (5%) compared to other antioxidants [97]. However, there were no significant effects noted when evaluating pregnancy rates.

### 4.3. Selenium

Selenium is an important molecule that helps produce selenoproteins, which are required for spermatogenesis and play an important role in protecting sperm during their transportation. In addition, selenium helps decrease oxidative stress by increasing the activity of glutathione peroxidase [119]. There are two randomized trials that found a benefit in sperm parameters following the supplementation of selenium [98,120]. However, a systematic review of 20 RCTs, including four studies specific to selenium, found no difference in sperm parameters or pregnancy rates [121].

### 4.4. Zinc

Zinc is the second most abundant mineral found in semen and usually originates from the prostate gland. Zinc plays an important role in testicular development and sperm maturation as it affects the stability of sperm chromatin [122]. A study showed that low levels of zinc were associated with lower sperm quality [123]. These findings were further noted in a randomized trial wherein zinc supplementation improved 18% of progressive sperm motility and 49% of sperm concentration [99]. However, to date, no study has shown a direct improvement in reducing DNA fragmentation following zinc supplementation.

### 4.5. Vitamins C, E, and D

Along with the supplements listed above, vitamins C and E are other antioxidants that can help regulate oxidative stress within spermatozoa [124]. In addition, vitamin D in its active form plays an important role in the synthesis of sex hormones and increasing intracellular calcium levels, which can improve male fertility [125]. A study led by Tadros et al. found that men with sufficient vitamin D levels had significantly more motile sperm [126]. These findings were also noted in a study conducted by Shahid et al., which noted a 20% improvement in motility and 5% improvement in sperm morphology in men with sufficient vitamin D levels [127]. A recent meta-analysis including five trials that evaluated the impact of vitamin D on male fertility showed a significant improvement in sperm motility and morphology but did not show a significant difference in sperm concentration [101]. Regarding the supplementation of vitamins C and E, Greco et al. demonstrated a significant decrease in DNA fragmentation levels among all patients studied after two months of treatment using a combination of vitamins C and E [100]. Nevertheless, these studies are limited as they have not demonstrated benefits in increasing the chance of live birth rates and are limited due to their lack of a control group and small sample size. In addition, a meta-analysis of 11 studies showed that vitamin C improved sperm motility and morphology, whereas vitamin E improved sperm motility and concentration [128]. In addition, both vitamin C and E improved pregnancy rates, but the quality of this evidence was low. Interestingly, while it is believed that vitamin C can regenerate oxidized vitamin E to enhance overall antioxidant capacity, when paired together, vitamins C and E did not improve sperm motility, concentration, or morphology [129]. Currently, no study reports live pregnancy rates following either vitamin C or vitamin D supplementation.

### 4.6. Lycopene

Lycopene is a carotenoid that can help scavenge ROS and decrease oxidative stress levels. A randomized controlled trial conducted by Williams et al. found that lycopene supplementation led to a 6% improvement in sperm morphology but did not lead to changes in sperm motility or levels of sperm DNA damage [102]. No study demonstrated an improvement in sperm motility or pregnancy rates following lycopene supplementation.

### 4.7. Folic Acid

Folate is a micronutrient that helps with DNA synthesis and also helps decrease levels of oxidative stress by inhibiting lipid peroxidation. A recent meta-analysis including eight randomized controlled trials evaluated the use of folate on sperm parameters [103]. They found that sperm motility significantly improved by 4%; however, there was no impact on sperm morphology, concentration, or pregnancy outcomes. In addition, another randomized trial using a combination of both zinc and folic acid supplementation demonstrated no change in sperm parameters, levels of sperm DNA methylation, and live birth rates [130,131].

### 4.8. Combination Therapy

Studies have also evaluated the benefit of combining multiple antioxidant supplements to improve sperm outcomes. The different antioxidant supplements used target different mechanisms to optimize fertility. A recent review article describes a novel approach for the combination of antioxidants by selecting them according to the intracellular mechanism they target throughout the three phases of sperm development [132]. This combination includes a long-chain omega-3 polyunsaturated fatty acid to target the mitotic phase, resveratrol to act as a ROS scavenger and prevent lipid peroxidation during meiosis, and lastly melatonin to decrease the production of ROS in the spermiogenesis phase [133].

The benefit of combining multiple antioxidants is challenged by the MOXI trial, which demonstrated that a combination consisting of vitamin C, vitamin E, selenium, carnitine, zinc, folic acid, and lycopene that was administered for three to six months did not yield a significant improvement in sperm parameters. Other studies have also shown that a combination of antioxidants did not improve sperm parameters or increase live-birth rates [134]. There is a hypothesis termed the antioxidant paradox wherein an inappropriate combination of antioxidant supplements may lead to detrimental effects on sperm parameters by increasing decondensation levels [135]. This term relies on the idea that sperm require a physiological concentration of ROS to perform certain reproductive functions, and; therefore, if this concentration of ROS is dysregulated due to an increase in reductive stress, it could lead to detrimental effects on one’s reproductive functions. This highlights the need to better understand the relationship between oral antioxidant supplements and oxidative stress levels to better identify the patients who would benefit from supplementation, as well as the optimal combinations and dosage of each supplement. Given the conflicting evidence, it is still unclear if antioxidant supplementation is beneficial for all patients. However, given the limited known harm, relative safety, and overall low cost, these can be recommended to help with idiopathic male infertility until more data clarifies this discrepancy [136].

After reviewing the evidence summarized in this review, we propose a generalized treatment regimen to allow for the standardization of treatment among men wishing to improve their fertility with the use of antioxidants. We do recognize that this combination regimen may not be ideal for all men suffering from various conditions and is limited due to its lack of formal evaluation in the clinical setting. The combination includes CoQ^10^, vitamin E, and vitamin C and is summarized in Table 3. However, unlike other combinations that do not always provide a rationale, this combination aims to target three distinct mechanisms to optimize sperm health including mitochondrial oxidative stress, lipid peroxidation, and preserving the integrity of sperm DNA. Given the mechanisms targeted by this treatment combination, this regimen would be recommended for men with a DFI greater than 30%, men with idiopathic oligo-asthenozoospermia, recurrent IVF failure, patients with poor-quality embryos, tobacco and e-cigarette smokers, obese men, and men who are post-varicocelectomy. There are multiple studies that support the use of these supplements as they have shown to improve sperm concentration, motility, morphology, and DNA fragmentation [8,95,100]. This combination is also complemented by the synergistic relationship between vitamin C and vitamin E to enhance one’s overall antioxidant capacity [129]. It is important to mention that this combination would be contraindicated among patients taking anticoagulants due to vitamin E’s ability to inhibit vitamin K-dependent clotting factors [137]. We would also not recommend this combination to known calcium oxalate stone formers and patients taking statins.

## 5. Therapeutic Applications

### 5.1. Current Treatment Protocols

The minimum recommended duration for antioxidant therapy is three months, corresponding to the complete spermatogenesis cycle [138]. This timeframe allows antioxidants to affect the entire development of sperm cells. Most studies demonstrate improvements in sperm parameters after at least three months of supplementation [95,97]. Presently, multiple antioxidant formulations exist, ranging from single agents to complex combinations. Evidence supporting various formulations varies considerably, as reported in the previously referenced systematic reviews. There is evidence demonstrating that combination therapies may offer synergistic effects through different mechanisms of action. For example, vitamin C can regenerate oxidized vitamin E, potentially enhancing overall antioxidant capacity [129]. However, the MOXI trial showed that even comprehensive formulations may not consistently show benefits across all populations of infertile men [108]. In addition, if these supplements are not well selected, this may lead to an antioxidant paradox and potentially decrease sperm parameters by increasing levels of sperm decondensation [135]. Currently, clinical guidelines specific to antioxidant supplementation remain limited. The American Urological Association and American Society for Reproductive Medicine joint guideline acknowledges that antioxidants may improve semen parameters but notes insufficient evidence for their routine use [11]. Similarly, the European Association of Urology recognizes the potential benefits of antioxidant supplementation but highlights the lack of conclusive data related to pregnancy outcomes [10]. Currently, most clinicians prescribe antioxidants empirically for men with idiopathic infertility or with evidence of increased oxidative stress as these have limited harm and cost to patients.

### 5.2. Personalized Treatment Protocols

As it was highlighted by Lahimer et al. in their recent review, the efficacy of antioxidant supplementation can vary significantly among studies due to many factors [12]. The variability in treatment responses suggests the need for personalized approaches. Several factors may influence one’s response to antioxidant therapy, including baseline oxidative stress levels, specific sperm abnormalities, and underlying pathologies. Measuring seminal oxidative stress levels prior to treatment may identify men most likely to benefit from antioxidant therapy. The MiOXSYS system offers a standardized method for measuring sORP to guide treatment decisions; however, its use is limited in men with oligozoospermia, the presence of a varicocele or leucocytospermia [69,71,72]. The CellROX^TM^ system is another promising technology that is widely accessible in centers without access to a flow cytometer. Similarly, measuring DNA fragmentation can identify men with sperm DNA damage who could benefit from targeted therapy that addresses these underlying issues [96]. In addition, the pattern of sperm abnormalities may guide treatment selection. Men with asthenozoospermia may benefit more from mitochondrial-targeted antioxidants like Coenzyme Q^10^ or carnitine, which improve energy metabolism [97,111]. Those with high DNA fragmentation levels may benefit more from vitamins C and E, which have shown efficacy in reducing DNA damage [100]. In addition, pre-existing underlying conditions should be considered when prescribing antioxidants. For example, men with varicocele have shown particular benefits from antioxidant supplementation, especially after varicocelectomy [139]. Men with metabolic syndrome, diabetes, obesity, or known smokers may require more intensive therapy due to systemic oxidative stress. Therefore, in these cases, antioxidant supplementation should be recommended in conjunction with other lifestyle changes [140]. Some studies also recommend measuring baseline serum antioxidant levels prior to administering them as supplements to ensure that patients will benefit from these antioxidants. However it is also important to mention that serum values may not always serve as reliable indicators of patients who will benefit from antioxidant supplementation [108]. This was noted in the MOXI trial wherein patients’ serum vitamin E, zinc, and selenium levels were not predictive of poor semen parameters or increased DNA fragmentation [132]. This can suggest that, while it is important to measure baseline serum antioxidant levels, this measurement may not help identify patients with increased oxidative stress who would benefit from antioxidant supplementation.

### 5.3. Combination with Other Fertility Treatments

Antioxidant therapy should be part of a comprehensive approach to male infertility treatment. Lifestyle modifications represent a crucial adjunct. Smoking cessation is particularly important among both tobacco and e-cigarette smokers as smoking increases oxidative stress and reduces the effectiveness of antioxidant supplements [24,26]. Smokers may require up to three times the standard dose to achieve comparable plasma levels to non-smokers [26]. Weight loss in obese men can also significantly improve hormonal parameters and oxidative stress markers [140]. Moderate exercise may enhance antioxidant capacity, while excessive exercise can increase oxidative stress [141]. Addressing sleep disorders is also important, as poor sleep quality correlates with increased oxidative stress and reduced testosterone levels [142]. Reducing exposure to environmental toxins may complement antioxidant therapy. Research suggests that mobile phone radiofrequency electromagnetic radiation may increase spermatozoa oxidative stress [14,15]. Limiting phone proximity to reproductive organs may help mitigate these effects. For men with varicoceles, combining antioxidant therapy with varicocelectomy may yield superior results compared to either treatment alone. Post-varicocelectomy supplementation can accelerate improvements in sperm parameters and DNA integrity [139]. While surgery addresses the anatomical cause of increased testicular temperature and oxidative stress, antioxidants help neutralize existing ROS during recovery. In the context of assisted reproductive technologies, antioxidant pretreatment of male partners may also improve outcomes. Several studies suggest that therapy prior to intrauterine insemination (IUI) or IVF may enhance success rates, particularly in cases with elevated sperm DNA fragmentation [143]. The optimal duration appears to be at least three months, corresponding to the spermatogenic cycle. Recent advances in sperm selection techniques for ARTs, such as magnetic-activated cell sorting or physiological intracytoplasmic sperm injection, may be synergistic with antioxidant therapy by reducing the impact of residual oxidative damage [144].

## 6. Challenges and Future Directions

### 6.1. Methodological Considerations in Antioxidant Research

Despite numerous studies, significant methodological limitations hamper definitive conclusions about efficacy. Heterogeneity among studies represents a major challenge, with variations in populations, formulations, dosages, treatment durations, and outcome measures [8]. Most studies focus on surrogate endpoints such as sperm parameters rather than clinical outcomes like pregnancy or live birth rates. While improvements in sperm parameters are encouraging, they do not necessarily translate to enhanced fertility [108]. Future research should prioritize clinical outcomes, particularly live birth rates.

The populations studied vary considerably, including men with idiopathic infertility, men with specific sperm abnormalities, or known causes of infertility. The heterogeneity of male infertility suggests that antioxidant efficacy may vary based on the underlying pathology, yet many studies do not adequately stratify participants. In addition, the lack of standardized oxidative stress measurement complicates research interpretation. Different studies use varying methods to measure oxidative stress, including direct ROS measurement, lipid peroxidation markers, DNA fragmentation, or total antioxidant capacity. Currently, the MiOXSYS system is used to measure levels of seminal oxidative stress [69]. However multiple studies have shown that it is limited in its ability to adequately identify patients that would benefit from the reductive stress generated by antioxidants. Therefore, standardized, clinically accessible assays would significantly help advance research to be able to identify the men who would benefit most from antioxidant supplementation [71]. Lastly, placebo effects must also be considered. In this review, many studies lack proper placebo controls, and the psychological impact of treatment may influence outcomes, particularly when trying to conceive naturally.

### 6.2. Dosing and Safety Considerations

Optimal dosing remains poorly defined, with significant variations in recommended doses across studies and antioxidant groups. For example, effective doses of Coenzyme Q^10^ range from 200 to 300 mg daily, while vitamin C doses range from 500 to 1000 mg daily [8,95]. The lack of dose-finding studies complicates clinical decision making. Antioxidant safety deserves greater attention. While generally considered safe, antioxidants may have adverse effects at high doses. Excessive supplementation might disrupt physiological ROS signaling, which plays important roles in sperm function [5]. Under certain conditions, antioxidants might act as pro-oxidants, potentially causing harm and creating an antioxidant paradox [145]. Regulatory oversight of dietary supplements, including antioxidants, varies globally and is often less stringent than pharmaceutical products. In the United States, the FDA regulates supplements under different rules than drugs. Manufacturers are not required to demonstrate efficacy before marketing, and quality control may be inconsistent [146]. This regulatory framework leads to variation in supplement quality, which may affect treatment outcomes. Drug interactions should also be considered when prescribing antioxidants. High-dose vitamin E may potentiate the effects of anticoagulants, while certain antioxidants may interfere with chemotherapy [137]. A comprehensive medication history should therefore be obtained before recommending a supplementation regimen.

### 6.3. Knowledge Gaps and Research Needs

Significant knowledge gaps remain in understanding the role of antioxidants in male fertility treatment. The mechanisms by which specific antioxidants improve sperm function remain incompletely understood. While general mechanisms of oxidative stress protection are known, the specific molecular pathways affected by different antioxidants warrant further investigation. Reliable biomarkers to predict antioxidant response represent a critical research need. Current approaches rely largely on empirical treatment, but predictive biomarkers could enable targeted therapy. Potential candidates include specific oxidative stress markers, genetic polymorphisms in antioxidant enzymes, or epigenetic modifications [147]. For example, several studies have examined levels of CoQ^10^, vitamin E, and vitamin D levels in both blood plasma and seminal fluid to correlate antioxidant concentration and levels of oxidative stress [148,149,150]. A promising avenue in biomarker research involves various -omics platforms, such as genomics, proteomics, and epigenomics, for diagnosing diseases. Given the high concentration of molecules within seminal fluid, these biomarkers can help serve as biomarkers for different etiologies of infertility and predict the success of ARTs [151].

Limited data exists on optimal combinations and dosing of antioxidants. While many commercial formulations contain multiple antioxidants, the scientific rationale for specific combinations is often lacking. Systematic evaluation of different combinations could identify synergistic formulations. The long-term effects of antioxidant therapy on male fertility and offspring health remain largely unexplored. Most studies focus on short-term outcomes, but potential epigenetic effects deserve investigation. Animal studies suggest that paternal antioxidant intake might influence offspring health through epigenetic mechanisms, but human data is limited [152]. The role of antioxidant therapy in specific clinical scenarios requires clarification. The optimal timing in relation to ART procedures, efficacy in men with genital tract infections, and role in men with systemic diseases all warrant further study. Larger, well-designed randomized controlled trials with clinical outcomes as primary endpoints will be necessary to establish definitive evidence for antioxidant efficacy in male infertility treatment. These studies should incorporate standardized oxidative stress measurements, clear inclusion criteria, adequate sample sizes, and long-term follow-up to provide conclusive guidance for clinical practice.

## 7. Conclusions

In our review article, we provide an overview of the vast information available regarding the role of antioxidants in minimizing ROS and optimizing male fertility. While this subject was extensively studied and has demonstrated the potential to serve as a supportive treatment for male infertility, we are not able to provide clear recommendations regarding the patients who would benefit most from antioxidant supplementation due to conflicting results noted in multiple trials. While there are several promising biomarkers being evaluated, there is a need for future studies to determine methods to better identify which patient profiles would benefit most from targeted antioxidant supplementation regimens. In addition, more studies are needed to evaluate the safety and long-term effects of antioxidants on reproductive outcomes.

## Figures and Tables

**Table 1:** Endogenous and exogenous antioxidant systems and their mechanisms.

	Antioxidant	Mechanism of Action
***Enzymatic Antioxidants***		
	*Superoxide Dismutase (SOD)*	Catalyzes the dismutation of superoxide radicals into hydrogen peroxide and molecular oxygen, offering frontline protection against ROS damage [66,67].
	*Catalase*	Converts hydrogen peroxide into water and oxygen, preventing its harmful accumulation. It is found in both sperm and seminal plasma [68].
	*Glutathione Peroxidase (GPx)*	Reduces hydrogen peroxide and lipid peroxides to non-toxic forms using reduced glutathione. It plays a critical role in protecting sperm mitochondria and nuclear material [69].
***Non-Enzymatic Antioxidants***		
	*Glutathione*	A tripeptide with strong ROS-neutralizing ability, glutathione protects sperm from oxidative damage and supports enzyme activity [70].
	*Coenzyme Q^10^*	CoQ^10^ functions in mitochondrial respiration and serves as a lipid-soluble antioxidant that enhances sperm motility and reduces oxidative injury [71].
	*Zinc and Selenium*	Both elements are essential for the structural and enzymatic integrity of sperm. Zinc stabilizes the sperm membrane and DNA, while selenium is a co-factor for glutathione peroxidase and sperm-specific selenoproteins [72,73].
***Vitamins***		
	*Vitamin C*	A water-soluble antioxidant concentrated in seminal plasma, vitamin C scavenges hydroxyl radicals and supports sperm viability [74,75].
	*Vitamin E*	A lipid-soluble antioxidant that interrupts lipid peroxidation in sperm membranes and works synergistically with vitamin C [76].
	*Vitamin D*	While primarily known for calcium regulation, vitamin D has emerging roles in sperm function and motility [77].
**Minerals**		
	*Selenium*	Essential for selenoprotein synthesis, selenium contributes to sperm structure and motility [78].
	*Zinc*	Vital for sperm chromatin stability, antioxidant enzyme activity, and ROS detoxification [79].
	*Copper*	A component of Cu/Zn-SOD; however, excess copper may enhance oxidative stress due to its redox cycling capacity [80,81].
**Other Antioxidant Compounds**		
	*Lycopene*	A carotenoid found in colorful fruits and vegetables; lycopene neutralizes ROS and helps preserve sperm DNA and membrane integrity [68].
	*Carnitines (*e.g., *L-carnitine)*	Support mitochondrial energy metabolism and sperm motility; their antioxidant action also reduces oxidative damage [82].
	*Omega-3 Fatty Acids (EPA, DHA)*	These polyunsaturated fats maintain membrane fluidity and integrity, offering both anti-inflammatory and antioxidant protection [72,74].

**Table 2:** Clinical effects of antioxidant supplementation on sperm parameters.

Supplement	Dose	Sperm Concentration	Sperm Motility	Sperm Morphology	DNA Fragmentation	Pregnancy Rate
Coenzyme Q^10^ [8,95]	100–300 mg/day	433%	350%	22%	9%	No improvement
Carnitine [96,97]	500–1000 mg/day	*	7%	5%	6%	No improvement
Selenium [8,98]	200 mg/day	No improvement	14%	No improvement	No improvement	No improvement
Zinc [8,99]	25–400 mg/day	49%	18%	*	No improvement	343%
Vitamin C [8,100]	500–1000 mg/day	No improvement	20%	12%	16%	*
Vitamin E [8,100]	400 mg/day	124%	36%	*	16%	571%
Vitamin D [8,101]	1400–50,000 IU/day	No improvement	5%	0.4%	*	*
Lycopene [8,102]	6–14 mg/day	28%	*	6%	No improvement	*
Folate [8,103]	0.5 mg/day	No improvement	4%	No improvement	No improvement	*

* Indicates that no data is available to measure the following parameter.

**Table 3:** Standardized recommended antioxidant treatment regimen.

Supplement	Dose	Justification
**Coenzyme Q^10^ [8,95]**	200–300 mg/day	Improves sperm motility by targeting the mitochondria. Data suggests an indirect impact on fertilization and embryonic development.
**Vitamin C [8,100]**	1000 mg/day	Reduces sperm DNA fragmentation, regenerates oxidized vitamin E. Studies show a synergistic effect with vitamin E on pregnancy rates.
**Vitamin E [8,100]**	400 UI/day	Protects against sperm lipid membrane peroxidation. An increase in pregnancy rates by 5 to 7 times has been reported in some studies.

## Abbreviations

The following abbreviations are used in this manuscript:

ROS	Reactive oxygen species
ARTs	Assisted reproductive techniques
ATP	Adenosine triphosphate
DFI	DNA fragmentation index
SCSA	Sperm chromatin structural assay terminal comet
TUNEL	Deoxynucleotidyl transferase dUTP nick end labeling
sORP	Static oxidoreduction potential
SCD	Sperm chromatin dispersion
IVF	In vitro fertilization
ICSIs	Intracytoplasmic sperm injections
SOD	Superoxide dismutase
CAT	Catalase
GPx	Glutathione peroxidase
GSH	Glutathione
CoQ^10^	Coenzyme Q^10^
PHGPx	Phospholipid hydroperoxide glutathione peroxidase
MOXI	Males, antioxidants, and infertility
IUI	Intrauterine insemination
